# The Royal Free Hospital

**Published:** 1893-10-14

**Authors:** 

**Affiliations:** British Embassy, Paris


### Editor's Letter-Box.

[Our correspondents are reminded that proxility is a great bar to publication, and that brevity of style and conciseness of statement greatly facilitate early insertion.]

### THE ROYAL FREE HOSPITAL.

Sir—As president of the Royal Free Hospital, Gray's Inn Road, I ask the favour of your allowing me to call public attention to its special need at the present time for £12,000 to enable the Committee to carry through the important work of completing the hospital buildings.

From the ruinous condition of the old front building, which was formerly a barrack for the Light Horse Volunteers, it became absolutely necessary to rebuild this portion of the hospital. The remaining portions were rebuilt some years ago, and are in excellent condition.

To carry out this work, which will provide much-needed isolation wards, casualty rooms, and dispensary, and to provide a new steam laundry, an expenditure of £20,000 has been incurred, towards which sum the Committee have raised £8,000, leaving a sum of £12,000 still urgently required.

The old front building has already been removed, and the work of reconstruction is now in active progress. Money is in consequence immediately required for the contractors. I therefore very earnestly solicit the help of those who have the means towards supplying the amount now needed, or to increase the current revenue by new annual subscriptions.

The Royal Free Hospital was the first general hospital in London to open its doors freely to the sick and suffering poor without letters of recommendation from governors. For 67 years it has adhered to this excellent system, and during that period over two millions of poor people have received within its walls the best medical and surgical advice and treatment.

During the past year 26,000 persons received its benefits either as in or out patients. The cost of maintenance was £10,551, whilst the ordinary income from all sources amounted to £5,118 only. The large deficit had to be met by utilising several considerable legacies which had fortunately been received during that year. In the previous year, 1891, there was, however, an absolute deficiency of £3,251, which had to be met by the sale of investments.

Contributions to either the building or general funds will be thankfully received by any member of the Committee; by the Hon. Treasurer, Edward Masterman, Esq., 27, Clement's Lane, E.C.; by the bankers, Messrs. Brown, Janson, and Co., 32, Abchurch Lane, E.C.; or by Mr. Conrad W. Thies, the Secretary, at the hospital.—I have the honour to be, Sir, your obedient servant, Dufferin and Ava.

British Embassy, Paris, October 10th, 1893.

